# Rapid identification and typing of *Yersinia pestis *and other *Yersinia *species by matrix-assisted laser desorption/ionization time-of-flight (MALDI-TOF) mass spectrometry

**DOI:** 10.1186/1471-2180-10-285

**Published:** 2010-11-12

**Authors:** Saravanan Ayyadurai, Christophe Flaudrops, Didier Raoult, Michel Drancourt

**Affiliations:** 1 Unité de Recherche sur les Maladies Infectieuses et Tropicales Emergentes: URMITE, UMR CNRS 6236-IRD 198, Faculté de Médecine, IFR48, Université de la Méditerranée, Marseille, France

## Abstract

**Background:**

Accurate identification is necessary to discriminate harmless environmental *Yersinia *species from the food-borne pathogens *Yersinia enterocolitica *and *Yersinia pseudotuberculosis *and from the group A bioterrorism plague agent *Yersinia pestis*. In order to circumvent the limitations of current phenotypic and PCR-based identification methods, we aimed to assess the usefulness of matrix-assisted laser desorption/ionization time-of-flight (MALDI-TOF) protein profiling for accurate and rapid identification of *Yersinia *species. As a first step, we built a database of 39 different *Yersinia *strains representing 12 different *Yersinia *species, including 13 *Y. pestis *isolates representative of the Antiqua, Medievalis and Orientalis biotypes. The organisms were deposited on the MALDI-TOF plate after appropriate ethanol-based inactivation, and a protein profile was obtained within 6 minutes for each of the *Yersinia *species.

**Results:**

When compared with a 3,025-profile database, every *Yersinia *species yielded a unique protein profile and was unambiguously identified. In the second step of analysis, environmental and clinical isolates of *Y. pestis *(n = 2) and *Y. enterocolitica *(n = 11) were compared to the database and correctly identified. In particular, *Y. pestis *was unambiguously identified at the species level, and MALDI-TOF was able to successfully differentiate the three biotypes.

**Conclusion:**

These data indicate that MALDI-TOF can be used as a rapid and accurate first-line method for the identification of *Yersinia *isolates.

## Background

The *Yersinia *genus is comprised of 15 species of γ-proteobacteria (http://www.bacterio.cict.fr/xz/yersinia.html) that are mostly harmless environmental organisms residing in soil and water [1]. Three *Yersinia *species are human pathogens, including *Yersinia pseudotuberculosis*, *Yersinia enterocolitica *and the plague agent *Yersinia pestis *[2-4]. While the two former species are food-borne pathogens responsible primarily for enteric infections, *Y. pestis *is an ectoparasite-borne species responsible for deadly plague [2]. Moreover, *Y. pestis *has been classified in the Centers for Disease Control's (CDC's) group A list of potential bioterrorism agents (http://www.bt.cdc.gov/agent/agentlist-category.asp). Thus, rapid and accurate methods of detection and identification are needed for the distinction of *Y. pestis *among other *Yersinia *species, as well as *Yersinia *organisms among other *Enterobacteriaceae *species.

Conventional methods for the phenotypic identification of *Yersinia *organisms such as biochemical profiling are time-consuming: they require the manipulation of huge quantities of potentially harmful pathogens and delay accurate identification beyond an appropriate time limit with respect to the medical management of patients and public health issues. PCR-based techniques [5] and real-time PCR assays reduce these delays to a few hours but require expertise and expensive reagents [6]. Furthermore, due to the natural instability of *Y. pestis *plasmids and chromosomal regions, molecular analysis may lead to false negative results when targeting specific genomic regions such as the 3a signature sequence [7-9]. Recognition of the F1 capsular antigen by several immunological techniques has been used for the rapid detection and identification of *Y. pestis *collected from patients with suspected infections [10] and from skeleton specimens from historical plague burial sites [11].

The identification of bacteria by matrix-assisted laser desorption/ionization time-of-flight (MALDI-TOF) mass spectrometry (MS) has recently emerged as a rapid and sensitive technology that provides protein profiles for the accurate identification of bacteria at the genus, species or sub-species level [12,13]. In microbiology, MALDI-TOF-MS has a number of potential advantages over other typing methods. Specimen preparation is relatively simple and can be carried out within minutes. Furthermore, the technique does not require any taxon-specific or costly materials such as antibodies. The workflow is simple and fast and can be standardized for most bacterial species. In addition, many of the procedures for sample preparation, data acquisition, and evaluation can be automated. Although MALDI-TOF-MS has been applied to several *Enterobacteriaceae *species, including *Y. enterocolitica *[14], it has not been described for other pathogenic *Yersinia *species, and only one report has dealt with the avirulent *Y. pestis *vaccinal strain EV 76 [15]. In this study, we assessed whether MALDI-TOF-MS could accurately identify all *Yersinia *species, including the three major biotypes of *Y. pestis*.

## Methods

### Bacterial strains

The following isolates were used to create an updated MALDI-TOF database comprising of 12 *Yersinia *species, except for *Yersinia similis*, *Yersinia aleksiciae *and *Yersinia entomophaga*: *Yersinia pestis *6/69M strain Orientalis biotype (kindly provided by Michel Simonet, Institut Pasteur, Lille, France), *Y. pestis *Nairobi-rattus (Antiqua biotype), *Y. pestis *14-47 strain Medievalis biotype (kindly provided by Joseph B. Hinnebusch, Rocky Mountain Laboratory, Hamilton, Montana and Florent Sebbane, Institut Pasteur, Lille, France), *Y. pestis *EV 76 (vaccine strain), six *Y. pestis *Medievalis isolates (5F1, 6b4, 8B7, 9F1, 5G5, 5B9) [16], *Y. enterocolitica *subsp. *enterolitica *CIP 8027, *Y. enterolitica *subsp. *paleartica *CIP 106945, *Y. enterocolitica *subsp. *enterocolitica *CIP 106676 (serotype 0:3), *Y. enterocolitica *subsp. *enterocolitica *CIP 8142 (serotype 0:9), *Y. enterocoIitica *subsp. *enterocolitica *CIP 101776, *Y. pseudotuberculosis *CIP 5585, *Y. frederiksenii *CIP 8029, *Y. intermedia *CIP 8028, *Y. kristensenii *CIP 8030, *Y. bercovieri *CIP 103323, *Y. mollaretii *CIP 103324, *Y. rohdei *CIP 103163, *Y. ruckeri *CIP 8280, *Y. aldovae *CIP 103162, and *Y. massiliensis *CIP 109351^T ^[17]. To test the identification abilities of MALDI-TOF, we used additional environmental and clinical isolates, including *Y. pestis *JHUPRI strain [18], two *Y. pestis *Orientalis biotype strains recently isolated from rodents in Algeria [19], ten *Y. enterocolitica *serotype O:9 (biotype 2) clinical isolates from feces in Nigeria (in collaboration with Joseph AE Okwori, Federal College of Veterinary and Medical Laboratory Technology, National Veterinary Research Institute, Vom, Nigeria), and one *Y. enterocolitica *strain isolated in our laboratory from stool. According to the French law, informed consent is not required from the individuals as far as the study concerns only microbiota and not the individuals themselves. The study of these isolates was approved by the Ethics Committee, Institute Fédératif de Recherche 48, Marseille, France. The *Yersinia *isolates were cultured on trypticase soy agar plates at 28°C for 2 days, and all *Y. pestis *isolates were cultured in a P3 laboratory in a biosafety level III cabinet with appropriate confinement protocols. Strains were identified by partial PCR amplification and sequencing of the *rpoB *gene [20]. *Y. pestis *typing was performed by multispacer sequencing typing (MST) using the spacers YP1, YP3, YP4, YP5, YP7, YP8, YP9, and YP10 as previously described [21]. The presence of plasmids in the *Y. pestis *isolates was evaluated by PCR analysis of plasmids pPst (pPCP1) [22], pFra (pMT1) [23] and pCD1 (pYV) via amplification of a 659-bp fragment of the *lcrV *gene using the forward primer 5'-GAATTGGTTCAGTTAGTCAA-3' and reverse primer 5'-AGATTACCCAACGCCCCGGT-3' and the following PCR program: an initial denaturation for 5 min at 95°C and 39 cycles of denaturation for 1 min at 94°C, annealing for 30 s at 56°C and extension for 1 min at 74°C, followed by a final extension for 10 min at 74°C. PCR products were analyzed in a 1.5% agarose gel containing 2 μg/ml ethidium bromide.

### Protocol for the inactivation of Yersinia organisms

To inactivate the bacteria, a 10-μl volume of 70% ethanol was added to the bacterial growth, vortexed in a biosafety level III cabinet and incubated at room temperature for 1 h. The effectiveness of the inactivation protocol for all samples was assayed prior to MALDI-TOF analysis by inoculating 50 μl of inactivated *Yersinia *suspension on a 5% sheep-blood agar plate and 50 μl into trypticase soy broth (AES, Rennes, France) and incubated them in parallel at 28°C for 7 days. The absence of any visible growth after 7 days of incubation was taken as evidence that the inactivation protocol was effective.

### MALDI-TOF-MS database

For each inactivated isolate, we deposited 1.5 μl of this suspension covered with 1.5 μl of matrix solution [saturated solution of alpha-cyano-4-hydroxycinnamic acid (α-HCCA) in 50% acetonitrile, 2.5% trifluoracetic acid] on a TP 384 target plate made of polished steel T F (Bruker Daltonics, Leipzig, Germany) and the matrix was then air-dried for 5 minutes. MALDI-TOF measurements were carried out using an Autoflex II mass spectrometer (Bruker Daltonics, Wissembourg, France) equipped with a 337-nm nitrogen laser. The instrument was calibrated every day using a reference *Klebsiella pneumonia*e isolate. Spectra were recorded in the positive linear mode (delay, 170 ns; ion source 1 (IS1) voltage, 20 kV; ion source 2 (IS2) voltage, 18.5 kV; lens voltage, 7 kV; mass range, 2-20 kDa). For each *Yersinia *sp. strain, the whole cell's protein profile was determined in triplicate. Each spectrum was obtained after 675 shots in automatic mode at variable laser power, and the time of acquisition was 30-60 seconds per spot. Automated data acquisition was performed with AutoXecute acquisition control software. The raw spectra obtained for each isolate were imported into MALDI BioTyper™ version 2.0 software (Bruker Daltonics) and analyzed by standard pattern matching (with default parameter settings) against the MALDI BioTyper™ database, an integrated part of the software (June 2008 version). Proteins between 3-15 kDa were identified by their m/z values. For each spectrum, up to 100 peaks were considered and compared to peaks in the database. The results were visualized with an intuitive graphical user interface. The peaks that were most similar (mass difference < 600 ppm) to the reference spectra appeared in green, while peaks with a mass difference > 600 ppm were shown in red or yellow. The 12 bacterial species exhibiting the most similar protein pattern to the strain under study were ranked by an identification score. The database (commercially available at Bruker Daltonics) was comprised of 3,025 MALDI-TOF profiles, including 42 strains of 11 *Yersinia *species, but lacking *Y. pestis*. We incorporated the profiles that we obtained for 39 different *Yersinia *isolates representative of 12 *Yersinia *species, including 13 *Y. pestis *strains, into this database. Every *Yersina *strain profile obtained in this study was also copied to a separate folder to form a new database in addition to the MALDI BioTyper™ database. The profiles were matched with the existing MALDI BioTyper™ database, and identification of the bacteria was carried out using MALDI BioTyper™ version 2.0.

### MALDI-TOF-MS identification

A total of 13 *Yersinia *isolates including 2 environmental *Y. pestis *Orientalis biotype isolates and 11 clinical isolates of *Y. enterocolitica *collected from feces were inactivated and blindly analyzed by MALDI-TOF-MS against the local updated database as described above. Identification scores were assigned using the following scoring parameters [13]: a score ≥ 1.9 indicated species identification; a score of 1.7-1.9 indicated genus identification; and a score < 1.7 indicated no identification. An isolate was considered to be correctly identified by MALDI-TOF when two of two spectra had a score ≥ 1.9. For organisms identified as *Y. pestis*, we further separated the protein profiles into three folders corresponding to each of the three biotypes. Using ClinPro Tools software, we analyzed the specific protein profile pattern for each biotype. ClinPro Tools software in-build, quick classifier and genetic algorithm analyses were used to differentiate the three *Y. pestis *biotypes. Quick classifier compares the average sprectum of the differentes classes in order to find the specific different peaks. The genetic algorithm creates a random peak list, changes the list ("mutation") and compares the discriminating capacity until obtaining the best list for discriminating classes.

### Reproducibility of MALDI-TOF-MS identification

In order to assess the reproducibility of MALDI-TOF-MS identification, every strain studied was tested in triplicate (i.e., on three different MALDI-TOF plates run on three different days from three different batches of culture). For every condition, 4 different spots were loaded on the MALDI-TOF plate, giving a total of 12 MALDI-TOF-MS protein profiles that were derived from each strain.

## Results

### Constituting a MALDI-TOF-MS Yersinia database

**Figure 1:**
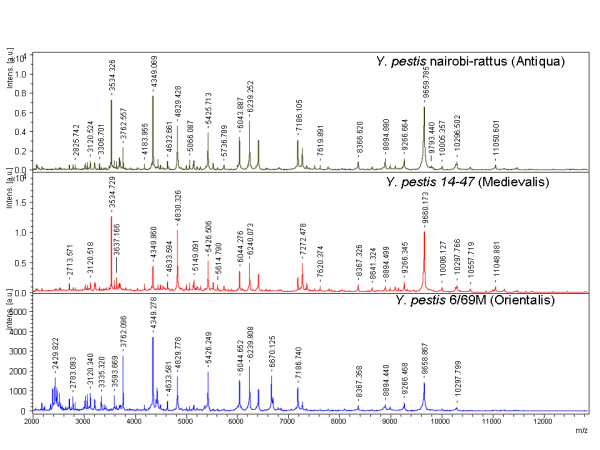
**Protein profile of the major *Y. pestis *biotypes generated by MALDI-TOF-MS. a.i., arbitrary intensity given by the software**.

**Figure 2:**
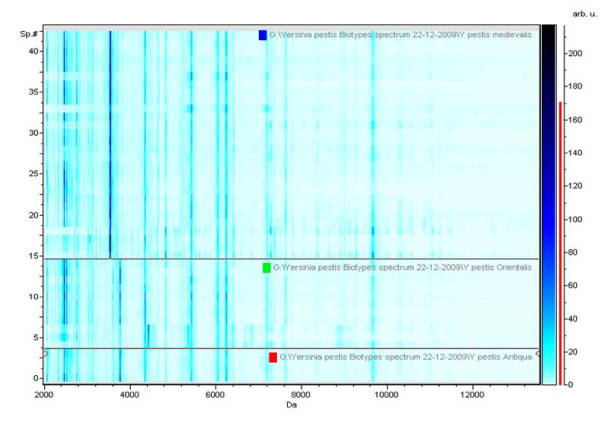
**Pseudo-gel representing the protein profile obtained after MALDI-TOF-MS analysis of *Y. pestis *organisms representative of the Antiqua, Medievalis and Orientalis biotypes**. arb.u., arbitrary unit - transcription for arbitrary intensity in the Bruker software; sp# is the numbers of the spectrum.

Accurate identification at the species level was confirmed for every isolate by partial sequencing of the *rpoB *gene. In addition, the presence of *Y. pestis *was confirmed by sequencing specific targets in each plasmid for each of the *Y. pestis *isolates used in this study. MST analysis discriminated the 13 *Y. pestis *isolates into 3 biotypes (Antiqua, Midievalis and Orientalis) with smaller variation in the number of alleles than previously reported [21]. The MST profile for the *Y. pestis *JHUPRI strain was most closely related to the Antiqua biotype but was atypical in that it contained spacer sequences from each of the three biotypes. The original MST profiles were deposited at the following website: http://ifr48.timone.univ-mrs.fr/MST_YPestis/mst. We observed no growth over 7 days for any of the *Y. pestis *isolates being studied after ethanol inactivation. MALDI-TOF protein profiles for the three main biotypes following 70% ethanol inactivation, including *Y. pestis *Antiqua (*Y. pestis *Nairobi-rattus), Medievalis (*Y. pestis *14-47), and Orientalis (*Y. pestis *6/69M) are shown in Figure 1. Figure 2 contains a pseudo-gel representing the protein profile for the three *Y. pestis *biotypes.

### MALDI-TOF-MS identification of Yersinia organisms

For the *Y. pestis *isolates, default identification against the Bruker database resulted in a false result of *Y. pseudotuberculosis *with an identification score > 2 in two of two cases. When the identification was performed using our local updated database, the isolates were correctly matched as *Y. pestis *in two of two cases with an identification score > 2.7, effectively identifying the isolates at the species level. The 11 *Y. enterocolitica *isolates were correctly identified as *Y. enterocolitica *with an identification score > 2. Further analysis of the *Y. pestis *isolates using ClinPro Tools software allowed us to assign them to a biotype, with the exception of the *Y. pestis *JHUPRI strain for which the unique MALDI-TOF profile did not match any of the three biotypes.

### Reproducibility of MALDI-TOF-MS identification

We obtained a unique MALDI-TOF profile for each of the 39 *Yersinia *isolates being studied: for each isolate, the 12 MALDI-TOF profiles derived from triplicate analysis were similar and yielded identical, accurate identification. A list of m/z values characteristic for *Y. pestis *is given in additional file 1.

## Discussion

Given that the MALDI BioTyper™ database contained 42 *Yersinia *profiles derived from 11 species but lacked the major pathogen *Y. pestis*, as well as the recently described species *Y. massiliensis *[17], we aimed to complete this database by deriving a MALDI-TOF profile for 12 species currently included in the *Yersinia *genus [17]. We obtained a unique MALDI-TOF profile for each of the *Yersinia *species used in this study. In each case, the species-specific profile did not match any of the 3,000 non-*Yersinia *profiles deposited in the MALDI BioTyper™ database, including those for closely-related enteric bacteria. We further observed that the MALDI-TOF profiles of *Yersinia *isolates yielded reproducible, accurate identification in agreement with previous reports indicating that varying culture conditions did not alter the accurate identification of enteric bacteria, including *Y. enterocolitica *[24,25]. We further established the proof of concept that MALDI-TOF-MS can be used for the identification of organisms belonging to any of the 12 species studied here. Blind MALDI-TOF analysis yielded an identification score ≥ 2 in 11 of 11 (100%) clinical isolates of *Y. enterocolitica *and in 2 of 2 (100%) of the *Y. pestis *isolates when compared to the updated database. An identification score ≥ 2 has been described as a valuable cut-off point for the accurate identification of bacterial isolates by MALDI-TOF analysis [13]. The ability to correctly identify isolates blindly indicates that MALDI-TOF is indeed a new and effective method for *Yersinia *species identification. This had already been established for *Y. enterocolitica *organisms but had not been described for the other pathogenic *Yersinia *species as the only report on *Y. pestis *included just the avirulent vaccinal strain EV 76 [15]. Notably, updating the database was crucial for the accurate identification of isolates as MALDI-TOF analysis of *Y. pestis *isolates using the original Bruker database resulted in false identification as *Y. pseudotuberculosis *with an identification score > 2. It has been previously observed that the quality of MALDI-TOF identification depends on the completeness and quality of the database used [13].

By using ClinPro Tools software as a second step, we were able to discriminate between the three main *Y. pestis *biotypes. The *Y. pestis *JHUPRI strain, however, was not identified as any of the three biotypes, in agreement with MST data indicating that it is an atypical strain [18]. This is consistent with previous observations that MALDI-TOF profiling is able to discriminate between various biotypes among other enteric species such as *Salmonella enterica *[26]. MALDI-TOF analysis can be supplemented with other state of the art techniques to ensure accurate genotyping of *Yersinia *isolates, including *Y. pestis. *While 16S rDNA sequencing and *rpoB *gene sequencing yield accurate identification of *Yersinia *organisms at the species level, [17,27,28] molecular typing of *Yersinia *organisms was done by MST [21], tandem repeat analysis [29-31], the detection of specific single-nucleotide polymorphisms [32], Enterobacterial Repetitive Intergenic Consensus PCR and Multilocus Sequence Analysis [27]. Mass spectrometry could be used for such determination thanks to emerging mass spectrometry-based methods for DNA analysis [33].

In this study, we inactivated all of the *Yersinia *organisms being studied even though such inactivation is not necessary for isolates belonging to species other than *Y. pestis *or when dealing with avirulent *Y. pestis *strains as previously reported [15]. We carried out an inactivation protocol to ensure that it did not significantly modify the results of the MALDI-TOF analysis. All of the inactivated *Yersinia *isolates with reference profiles in the Bruker database were unambiguously identified using this database, indicating that the inactivation protocol did not significantly interfere with MALDI-TOF identification. We hypothesized that a previously published inactivation protocol based on the incubation of *Y. pestis *with Tween and formalin, an agent that denatures proteins, may significantly modify the peptide profiles of isolates and affect their identification [33]. As expected, the inactivation of *Yersinia *by incubation with 80% TFA for 30 minutes as previously proposed for vegetative cells and spores did not yield interpretable profiles (data not shown) [34]. The protocols for ethanol inactivation tested in this study took 1 hour to inactivate the organisms; however, this step may be omitted if the mass spectrometer is used in a biosafety level 3 laboratory, although this was not the situation in our study. MALDI-TOF-MS identification can be completed in less than 10 minutes, less time than is required for Gram staining analysis [13]. The mass spectra of whole cells provide a snapshot of different protein compositions of individual microbial strains and thus constitute strain-specific suites of biomarkers. MALDI-TOF identification, therefore, is a more rapid technique for the identification of *Yersinia *isolates. Previously, only detection of the F1 capsular antigen using hand-held kits had proven to be an excellent bench-top technique for the rapid identification of *Y. pestis *[35]. In a comparative analysis, detection of the F1 antigen was highly specific and sensitive enough to positively identify ten of ten *Y. pestis *isolates from various countries [35]. The delay in identification varies from 20 minutes for an immunochromatographic test [10] to 2 hours for immunofluorescence microscopy [35]: however, the most accurate immunochromatographic test is not yet commercially available [35]. Given that it is based on the analysis of dozens of phenotypic characteristics into a unique profile, MALDI-TOF identification is less prone to variability and false negative results than phenotypic identification based on only one phenotypic characteristic such as the *Y. pestis *F1 capsular antigen. The F1 capsular antigen is plasmid-encoded and might be unstable; thus, it is risky to assume correct identification based on just one phenotypic trait. False negative results have been reported in cultures incubated at temperatures less than 37°C as this antigen is expressed by *Y. pestis *only between 33-37°C [1]. The same holds true with regard to direct detection of the F1 capsular antigen in specimens that have been refrigerated for more than 30 hours [1]. Therefore, MALDI-TOF identification appears to be the most rapid test for the accurate identification of *Y. pestis *and other *Yersinia *species organisms.

## Conclusion

In conclusion, MALDI-TOF can be used as a first-line method for the accurate identification of *Yersinia *organisms using an updated database that includes profiles of all *Yersinia *species. MALDI-TOF identification is the most rapid identification tool and is available at a low cost (< $1 per identification). *Y. pestis *should be added to the list of bioterrorism agents such as *Bacillus anthracis *that are readily identifiable by MALDI-TOF-MS [36,37].

## Authors' contributions

AS, DR and MD designed the experiments and wrote the paper. AS and CF performed the experiments. DR and MD coordinated the project. All authors have read and approved the manuscript.

## Supplementary Material

Additional file 1 **List of m/z values of MALDI-TOF peaks characteristic for *Y. pestis*: m/z values are given in the first column, the signal/noise (S/N) ratio is given in the second column**. Click here for file
